# The Hexameric Structures of Human Heat Shock Protein 90

**DOI:** 10.1371/journal.pone.0019961

**Published:** 2011-05-25

**Authors:** Cheng-Chung Lee, Ta-Wei Lin, Tzu-Ping Ko, Andrew H.-J. Wang

**Affiliations:** 1 Institute of Biological Chemistry, Academia Sinica, Taipei, Taiwan; 2 Core Facility for Protein Production and X-ray Structural Analysis, Academia Sinica, Taipei, Taiwan; Griffith University, Australia

## Abstract

**Background:**

The human 90-kDa heat shock protein (HSP90) functions as a dimeric molecular chaperone. HSP90 identified on the cell surface has been found to play a crucial role in cancer invasion and metastasis, and has become a validated anti-cancer target for drug development. It has been shown to self-assemble into oligomers upon heat shock or divalent cations treatment, but the functional role of the oligomeric states in the chaperone cycle is not fully understood.

**Principal Findings:**

Here we report the crystal structure of a truncated HSP90 that contains the middle segment and the carboxy-terminal domain, termed MC-HSP90. The structure reveals an architecture with triangular bipyramid geometry, in which the building block of the hexameric assembly is a dimer. In solution, MC-HSP90 exists in three major oligomer states, namely dimer, tetramer and hexamer, which were elucidated by size exclusion chromatography and analytical ultracentrifugation. The newly discovered HSP90 isoform HSP90N that lacks the N-terminal ATPase domain also exhibited similar oligomerization states as did MC-HSP90.

**Conclusions:**

While lacking the ATPase domain, both MC-HSP90 and HSP90N can self-assemble into a hexameric structure, spontaneously. The crystal structure of MC-HSP90 reveals that, in addition to the C-terminal dimerization domain, the residue W320 in the M domain plays a critical role in its oligomerization. This study not only demonstrates how the human MC-HSP90 forms a hexamer, but also justifies the similar formation of HSP90N by using 3D modeling analysis.

## Introduction

Heat shock protein 90 (HSP90) is an ATPase-dependent chaperone and the molecular chaperone functions as a dimer. HSP90 is responsible for managing protein folding and quality control in the crowded environment inside the cell. It participates in activating and stabilizing more than 200 “client” proteins involved in post-translational folding, protein stability, activation and maturation of cellular proteins, which are essential to cell-cycle control and signaling. HSP90, HSP70 and co-chaperones form a dynamic complex known as the HSP90 dynamic machine [1], which is regulated by co-chaperones and post-translational modification, e.g., phosphorylation, nitrosylation and acetylation for client protein interaction and ATPase activity. The yeast HSP90-Sba1 complex structure provides a view of HSP90 in the ATP-bound state, demonstrates the conformational changes in the N-terminal domain and reveals how the co-chaperone Sba1 recognizes the “closed” state of HSP90 dimer, that confirms the ATPase-coupled molecular clamp mechanism of HSP90 chaperone [2]–[3]. Many oncoproteins are HSP90 client proteins, including EGFR, AKT, MMP2 and BCR-ABL. They depend on its protein folding machinery to avoid misfolding and degradation in cancer cells. HSP90 inhibition offers a great promise in the treatment of a wide variety of solid and haematological malignancies. Therefore, HSP90 has been a target for anticancer drugs, and several classes of compounds have been and are being developed to modulate its activity for therapeutic benefit [4]–[5].

The HSP90 proteins are highly conserved and five human isoforms have been identified, including two cytosolic isoforms HSP90α and HSP90β, a glucose-regulated protein (GRP94) in the endoplasmic reticulum, a tumor necrosis factor receptor-associated protein 1 (Hsp75/TRAP1) in the mitochondrial matrix, and a newly discovered isoform HSP90N [6]. These isoforms exhibit different domain structure and cellular location, and may have different client protein substrates [4], [7]. Recent studies also indicate that many types of cells express HSP90 on the cell surface and secrete HSP90 into the extracellular space to carry out important extracellular functions [1], [8].

The conserved HSP90 structure consists of three domains: an N-terminal (N) domain that contains the co-chaperone binding motif and an ATP and drug binding site that binds the natural compounds geldanamycin and radicicol; a middle (M) domain that is responsible for binding to co-chaperone and client proteins; and a C-terminal (C) domain that contains a dimerization motif, a second drug-binding site, and a conserved MEEVD pentapeptide at the C-terminus, which is recognized by the co-chaperone HSP70/HSP90 organizing protein (Hop) [9]. This C domain was predicted to contain a second nucleotide-binding site, which has been shown to bind to novobiocin, epilgallocatechin (ECGC) and taxol [10]. However, neither the apo-form crystal structure nor any complex structure has been reported for the M and C domains of human HSP90. The isoform HSP90N is a plasma-membrane-associated protein in poorly-differentiated colorectal cancers with metastasis [11]. Specifically, HSP90N lacks the N-terminal nucleotide-binding domain which is replaced by a 30-residue-long hydrophobic motif, but it shares the 509 amino acids in the C-terminal region with HSP90α [6], [12]–[13].

HSP90 exists as a homodimer, and the dimers tend to associate into tetramers, hexamers, and even higher oligomers. It has been reported that the oligomeric forms of HSP90 are present in the cytosol of mammalian cells [14]. The heat-induced HSP90 oligomer can bind to its substrates and prevent their irreversible aggregation, and the self-oligomerization of HSP90 may have a pivotal role in protecting cells from thermal damages by its chaperone function [15]–[16]. HSP90 oligomerization can be induced by heat treatment, the presence of nonionic detergents or addition of divalent cations, such as magnesium and calcium. The heat-induced oligomerization can be inhibited by ATP and geldanamycin, both bind to the same pocket in the N-terminal domain [15], [17]–[18]. HSP90 association into tetramer, hexamer and dodecamer forms, as induced by Mg^2+^ ion, demonstrated that the building block for its oligomerization is a dimer. In particular, the hexamer with a cozy nest-shape was obtained by negative staining TEM tomography [18]. In addition, the divalent cation-induced oligomerization is accompanied by the instantaneous loss of its molecular chaperone function [19]. The functional roles of HSP90 oligomers in the chaperone cycle are not clearly understood, therefore the oligomerization process and the oligomeric structures are studied here. In this study, we determined the crystal structure of a human HSP90 that contains the M and C domains (MC-HSP90) in a hexameric assembly. The oligomerization sites are located in the M domain and also involve the dimerizing C domain. The hydrodynamic properties of MC-HSP90 and HSP90N in solution were also examined. The results clearly showed that MC-HSP90 exists in three major states: dimer, tetramer and hexamer. HSP90N also showed a dimeric and two higher oligomer states as did MC-HSP90 under similar conditions. The hexameric structure of MC-HSP90 provides a clue to how the possible oligomers of HSP90N establish their functional architecture.

## Results

### Crystals and Structures of MC-HSP90

**Figure 1:**
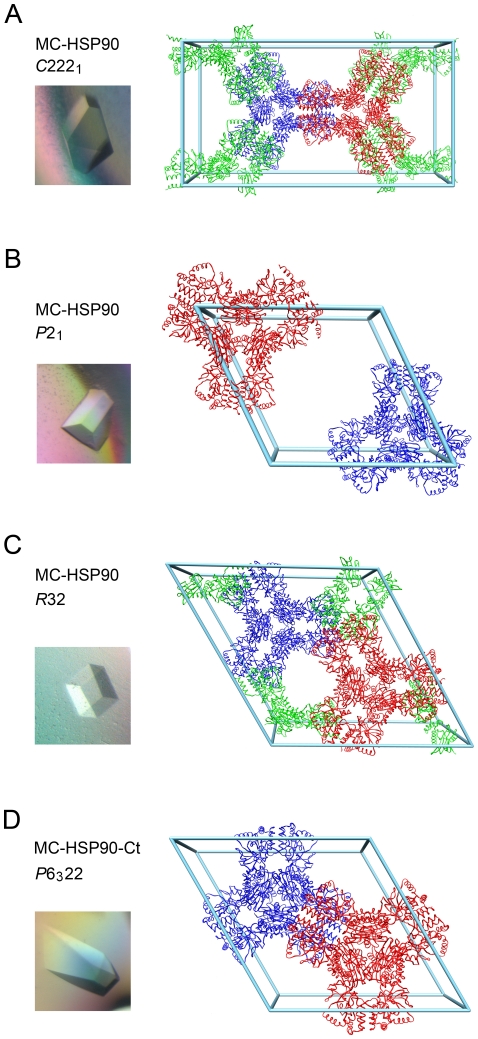
Crystal packing of MC-HSP90 molceules. The pictures of the (A) orthorhombic, (B) monoclinic, (C) rhombohedral, and (D) hexagonal crystals are shown on the left side. The unit-cell contents of the corresponding crystals are shown on the right. All of these crystals contain structurally similar hexamers, which are shown in three different colors and have a variety of arrangements in the unit cells. Although the green models appear to be disintegrated, they indeed form intact hexamers when associated with their symmetry-related mates from the neighboring unit cells. Only the orthorhombic and the hexagonal crystals (A and D) show some resemblance in packing, but they contain proteins with different C-termini.

The N-terminal truncated HSP90 (MC-HSP90, residues 293–732) containing the middle segment and carboxy-terminal domain was crystallized in three different forms, *C*222_1_, *P*2_1_ and *R*32. A fourth crystal form of *P*6_3_22 was obtained using a modified MC-HSP90-Ct (residues 293–697) in which the C-terminal 35 residues were also truncated. The crystals were obtained using different protein preparations or under different crystallization conditions. For the *C*222_1_ and *P*2_1_ crystals, obtained under similar conditions, the protein solution was pre-incubated with *cis*-dichloro(ethylenediamine)platinum(II) (*cis*-DEP) and cisplatin, respectively, before crystallization setup. The *R*32 crystals were obtained by using an additive solution in the crystallization drops. The *P*6_3_22 crystals were grown under low pH conditions, also with an additive. (See Materials and Methods for details.) All crystal structures were determined by molecular replacement (MR) method, but only the *C*222_1_ and *P*2_1_ crystals were refined to 3.0 Å and 3.05 Å resolutions, respectively (Table 1), with three and six molecules of MC-HSP90 per asymmetric unit. The *R*32 and *P*6_3_22 crystals have one molecule per asymmetric unit. The protein molecules in all crystal forms show a hexameric architecture with triangular bipyramid geometry in the protein assembly, and in the latter two forms (*R*32 and *P*6_3_22) the molecular triad and dyads are further expressed as crystallographic symmetry elements (Figure 1). If the protein solutions were not pre-incubated with the platinum compounds before the crystallization drop setup or the additive solution were included only in the drops, the protein could still be crystallized but with poor crystal quality; those crystals only diffracted to about 6 Å resolution and the structure could not be determined.

**Table 1:** Data collection and refinement statistics^a^.

Additive compounds	*cis*-DEP	cisplatin
Data collection		
Wavelength (Å)	1.0	1.0
Space group	*C*222_1_	*P*2_1_
Cell dimensions (Å, °)	*a* = 162.70, *b* = 304.55, *c* = 87.62,	*a* = 157.90, *b* = 90.87, *c* = 167.09,
		*β* = 115.85
Resolution (Å)	30-3.0 (3.11-3.0)	30-3.05 (3.16-3.05)
Observed reflections	186,922	291,396
Unique reflections	43,634	82,281
*R*_sym_ (%)	5.8 (45.1)	5.8 (36.2)
*I*/σ(*I*)	17.9 (3.5)	19.6 (5.1)
Completeness (%)	99.6 (99.3)	98.8 (99.4)
Redundancy	4.3 (4.2)	3.5 (3.5)
Z	3	6
Refinement		
Resolution (Å)	30-3.0	30-3.05
No. of reflections *R*_work_/*R*_free_	39,180/2,185	72,417/4,017
*R*_work_/*R*_free_ (%)	20.9/25.2	20.4/25.9
No. of atoms		
Protein	9,197	17,827
SO_4_	15	30
Water	133	281
Avg B factor (Å^2^)		
Protein	90.7	82.3
SO_4_	138.6	124.3
Water	92.0	85.9
RMSD		
Bond lengths (Å)	0.012	0.012
Bond angles (°)	1.405	1.399
Ramachandran statistics (%)^b^		
Most favored	88.6	89.9
Additionally allowed	9.6	9.2
Generously allowed	1.5	0.5
Disallowed	0.4	0.4

a Values corresponding to the highest resolution shell are shown in parentheses.

b The stereochemistry of models were validated with PROCHECK.

The refined models of MC-HSP90 in the two different space groups of *C*222_1_ and *P*2_1_ contain nine independent polypeptide chains in total. The N-termini start at residues 294–297, and the C-termini stop at residues 697–699, depending on the corresponding electron densities for the individual subunits. The residue 293 and the last 33 residues, plus the His-tag, were not observed, probably because they were highly flexible. Other disordered regions were located in residues 349–357, 394–404 and 611–629. Three molecules had an additional disordered region in residues 558–613, which also showed high temperature factors in the other molecules. In an attempt to obtain better crystals, we constructed a C-terminal tail truncated MC-HSP90-Ct, but the resulting *P*6_3_22 crystals did not show any improvement in the diffraction resolution. The *R*32 crystal showed additional electron densities for most of these regions, but they could not be modeled with certainty due to the poor diffraction resolution. These two structures could only be refined to 3.5 Å resolution, yielding *R* values of about 30% and *R*_free_ of about 35%.

**Figure 2:**
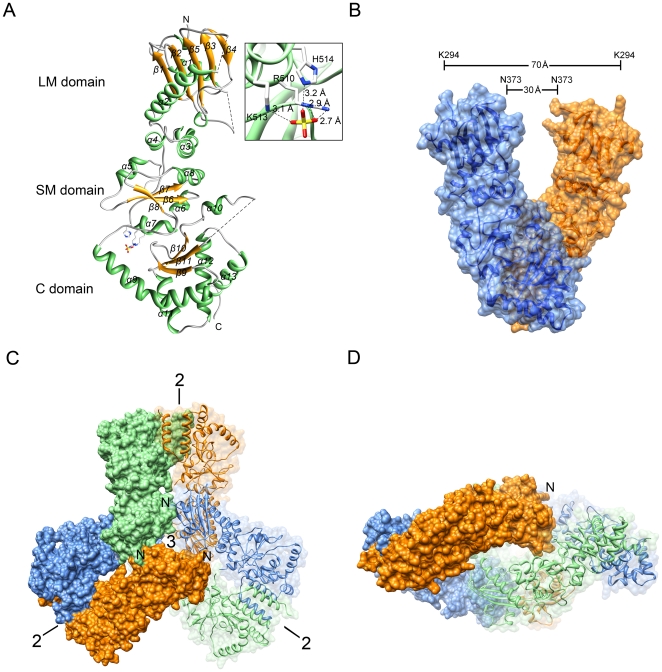
Overall structure of MC-HSP90. (A) Ribbon representation of the MC-HSP90 monomer with a bound sulfate. The large middle (LM), small middle (SM) and C-terminal domains (C) are indicated. The inset figure shows a sulfate bound to R510, K513 and H514. (B) Parallel homodimer of MC-HSP90. Two protomers (blue and orange) extend from its C-terminal dimerization domain with “Twisted V” shape and are shown in ribbon and translucent surface. The inner and outer distances between the open ends are indicated. (C) (D) Top view and side view of the hexameric MC-HSP90 structure. Six MC-HSP90 molecules assemble into a dextral hexamer. The N-termini are located on the top and bottom of the hexamer with 3-fold symmetry. Each protomer is colored in separate colors (blue, green and orange) and half of hexamer is shown with translucent surface.

**Figure 3:**
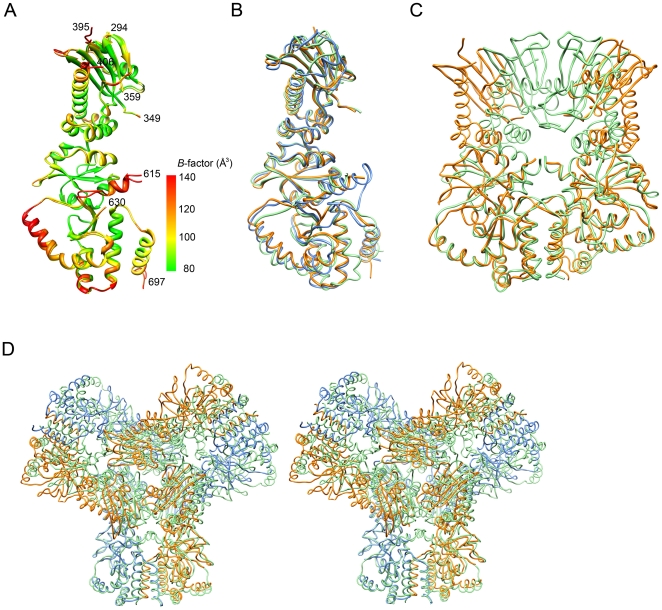
Comparison of HSP90 structures. (A) Disordered regions of MC-HSP90. The protomers A and B in the *C*222_1_ crystal are superimposed with RMSD of 0.637 Å between 367 Cα atom pairs. The residue number of N- and C-termini and the terminal ends of disordered loops in protomer A are indicated. The protein models are presented with ramped colors according to the *B* values. The start of the curved α-helix (α9) and the extended arm (α10) at C domain show high temperature factors. (B) The monomer structure of human MC-HSP90 (orange) is superimposed on the MC domains of closed-form yeast HSP82 (green) and open-form canine GRP94 (blue). (C) The dimer structure of human MC-HSP90 (orange) is superimposed on the MC domains of close-form yeast HSP82 (green). (D) Stereo view of superposed hexameric structures. The human MC-HSP90 hexamer and the N-terminal truncated yeast HSP82 (PDB: 2CGE) hexamer are superimposed. The protomers of Human MC-HSP90 dimer are colored in blue and orange, respectively, and six protomers of yeast HSP82 are colored in green. See Video S1 for more comprehensive aspects.

In the current structure, each molecule folds into three major domains (Figure 2A): a large middle (LM) domain (residues 293–469), a small middle (SM) domain (residues 470–547), and the C-terminal (C) domain (residues 548–732). The structure of the LM domain starts with a 3_10_-helix, which is followed by a three-layer architecture of α-β-α sandwich and a helical coil. It encompasses two disordered loops comprising residues 351–358 and 396–404. The SM domain is also folded into an α-β-α sandwich architecture, and contains a sulfate-binding site. The sulfate was hydrogen bonded to the side chains of R510, K513 and H514 within helix α7, which is close to the C domain (Figure 2A). The C domain begins with an amphipathic loop, which shows high *B*-values (Figure 3A), and contains a curved α-helix, a three-stranded β-sheet, a three-helix coil and an extended disordered arm between helix α10 and strand β11 (residues 617–629). Beyond residue 697, the structure is disordered.

### Structure-based Sequence Alignment

**Figure 4:**
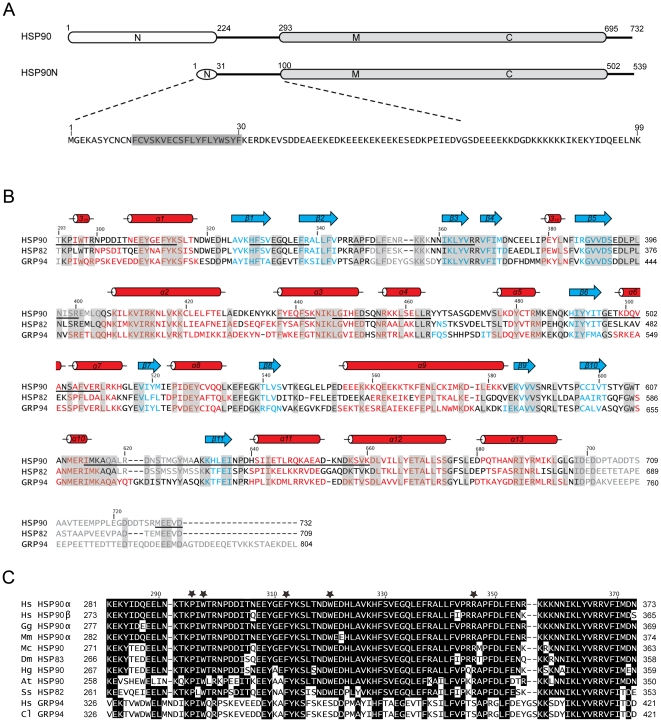
Sequence alignment. (A) Schematic representation of human HSP90 and HSP90N. The functional domains (N, M, and C) and the residue number are indicated. The N-terminal sequence (residues 1–99) of HSP90N is shown below and a predicted transmembrane helix is highlighted in gray. (B) Structure-based sequence alignment of the MC domains of human HSP90, yeast HSP82 (PDB: 2CG9) and canine GRP94 (PDB: 2O1V). Residues of α-helices, β-strands, and disorders are shown in red, blue, and gray, respectively, and those of identical residues are highlighted by gray boxes. The secondary structural elements according to the human HSP90 structure are shown above the sequences, and the bars under the HSP90 sequence indicate the segments which have been identified by MALDI-MS analysis. Two extra Ala residues at the N-terminus and the C-terminal His-tag also have been identified, but are not shown in the figure. (C) Part of the M domain sequence from human HSP90 is aligned with other 90-kDa heat shock proteins of *Homo sapiens* (Hs), *Gallus gallus* (Gg), *Mus musculus* (Mm), *Macrocentrus cingulum* (Mc), *Drosophila melanogaster* (Dm), *Heterodera glycines* (Hg), *Arabidopsis thaliana* (At), *Saccharomyce scerevisiae* (Ss) and *Canis lupus* (Cl) for the regions near the W320 binding site. The residues involved in M domain interaction in the human MC-HSP90 hexamer are marked with black stars.

Two determined 90-kDa chaperone structures, yeast HSP82 and canine GRP94 [3], [20] were chosen for comparison with the middle and C-terminal domains of human HSP90. Sequence alignment shows that human MC-HSP90 and yeast MC-HSP82 have about 60% identity in 438 residues. The core domain and loops regions are highly conserved, but the C-terminal extension is variable (Figure 4B). The MC-HSP90 monomer superimposes well on the MC domain of yeast HSP82 (PDB: 2CG9) and yields an rmsd of 0.91 Å for 219 Cα atoms. Human MC-HSP90 and canine GRP94 (PDB: 2O1V) have only about 49% identity in 414 residues of the MC domain. When the domain structures are superimposed, the rmsd value is 1.02 Å for 241 Cα atoms. Both yeast HSP82 and canine GRP94 show structural similarity to the human HSP90 in the MC domain (Figure 3B).

In the absence of the N-terminal ATPase domain, co-chaperone and client proteins, MC-HSP90 structure contains several disordered regions, which correspond to four functional loops: an amphipathic loop (residues 347–360), a middle segment catalytic loop (residues 395–406), an extended loop (residues 616–630) and the C-terminal tail (residues 698–732). To confirm the presence of disordered loops and C-terminal tail in the crystal, the *C*222_1_ crystal was harvested and dissolved for in-gel trypsin digestion and MALDI-MS analysis. In this experiment, the N and C-terminal tail segments and the residues comprising the amphipathic loop, as well as the extended loop, can all be identified in the MASS spectra. However, in the catalytic loop, only the residues 395–400 could be identified (Figure 4B) by our experimental procedure. In the yeast HSP82/Sba1 complex structure, the equivalent regions of these disordered segments were not observed either, except for the middle segment catalytic loop, because it contained the catalytic residue R380 (equivalent to R400 of human HSP90) bound to a nucleotide, which stabilized the loop [3].

### Structural Features and Comparison

It has been proposed that HSP90 functions as a dimer, and the C-terminal domain is involved in the major dimeric interactions [21]. The previously determined 90-kDa chaperone structures also supported this [3], [20]. Here, in the truncated MC-HSP90 structure, the protein subunits are arranged into a parallel homodimer with a left-handed helical twist. Each protomer extends from its C-terminal helix coil at the central axis of the dimer to form a “Twisted V” shape, which corresponds to the “open” state of HSP90 structure. In the open end, the distance is about 30 Å between the Cα atoms of N373 from the two protomers, and it is about 70 Å between the two N-terminal residues K294 (Figure 2B). Helices α12 and α13 in the C-terminal domain of two dyad symmetry related protomers are folded into a four-helix bundle. The interaction interface consists of both hydrophobic and hydrophilic contacts, which is the most important stabilizing element for HSP90 dimerization. A surface area of about 1,267 Å^2^ on each protomer is buried by its counter protomer upon dimer formation (Figure 2C). Furthermore, when the dimers are superimposed for the three-helix coil of C domain (Figure 3C), the MC-HSP90 dimer shows an open-form in contrast to the close-form yeast HSP82, differing from each other by a large sway of the protomers. The loop region between the SM and C domain, the “curved helix” (α9) and the extended loop include helix α10 have large conformational changes, which result in the domain shift of M domain. The flexible conformation of the curved helix α9 and the connection loop between M and C domain allow the middle domain to have a large domain shift for the alternation between open and closed conformations of the HSP90 clamp.

### Quaternary Structure of MC-HSP90

**Figure 5:**
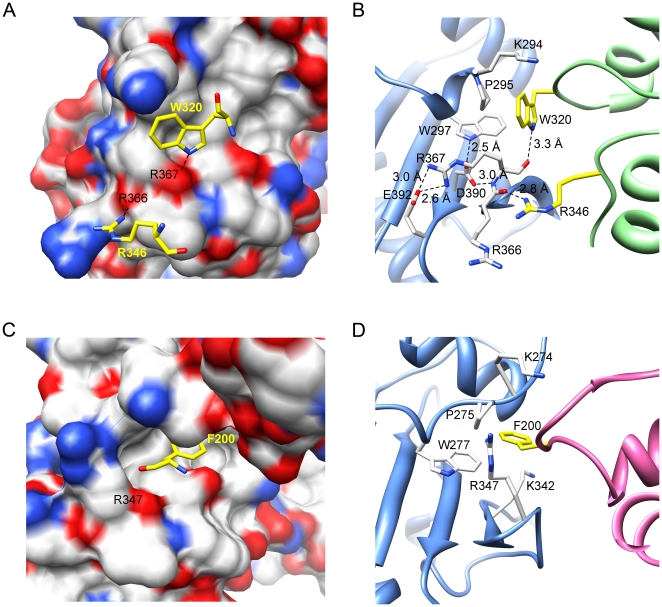
Protomer interactions in the M domain. (A) A protomer is shown as an electrostatic surface that contains a concave cavity for residues W320 and R346 packing with the neighboring protomer. (B) The major contacts of M domain between protomers, colored in blue and green, are shown with stick models. (C) The corresponding region of M domain in the full-length yeast HSP82 (PDB 2CG9) is shown as a surface. F200 from the N domain appears to take over the place of W320. (D) The contacting M and N domains in yeast HSP82 are colored green and pink, and the corresponding interactions are depicted with stick models. T273, F292 and Y344 (not shown) form part of the hydrophobic pocket but are not involved in direct contact.

The *P*2_1_ crystal has three open-form MC-HSP90 dimers in an asymmetric unit, which assemble into a triangular bipyramid architecture being arranged in a right-handed fashion around a pseudo 3-fold axis (Figure 2C and D). The other crystals, the *C*222_1_ and *R*32 crystals of MC-HSP90 and the *P*6_3_22 crystal of MC-HSP90-Ct have fewer than six molecules in an asymmetric unit, but they also contain this kind of hexameric assembly in the crystals, suggesting that the hexamer is not just a result of stabilization by favourable crystal lattice contacts. A top view of the hexameric structure shows a trefoil-like shape, with the N-terminal end of the monomer facing up and the C-terminal end directing radially away from the center. The top view shows a compact arrangement of the three monomers, which belong to three different dimers. The contact between the monomers around the 3-fold axis is facilitated by the N-terminal part of LM domain. One subunit is in contact with both 3-fold symmetry-related subunits via the inter-dimer interface, that buries about 531 Å^2^ surface area on a monomer for the hexamerization, the major contact region is shown in Figure 5. The side chain of residues W320 and R346 pack against a concave surface of the neighbouring protomer (Figure 5A). The W320 side chain is accommodated in a hydrophobic pocket formed by K294, P295 and W297, and also forms a hydrogen bond to the main chain oxygen atom of R367 of the neighboring protomer. The side chain of R346 is hydrogen bonded to the main chain oxygen atom of R366 (Figure 5B). This interaction positions the LM domains about the center of the complex, with N-terminnal end protruding around the central 3-fold axis, three facing up and three facing down. Viewed from the side, the hexameric structure appears to have an elliptic shape. The middle domain is packed against one another in the central core, and the C-terminal domain also contacts one another at the rim.

### Oligomer States of MC-HSP90 in Solution

**Figure 6:**
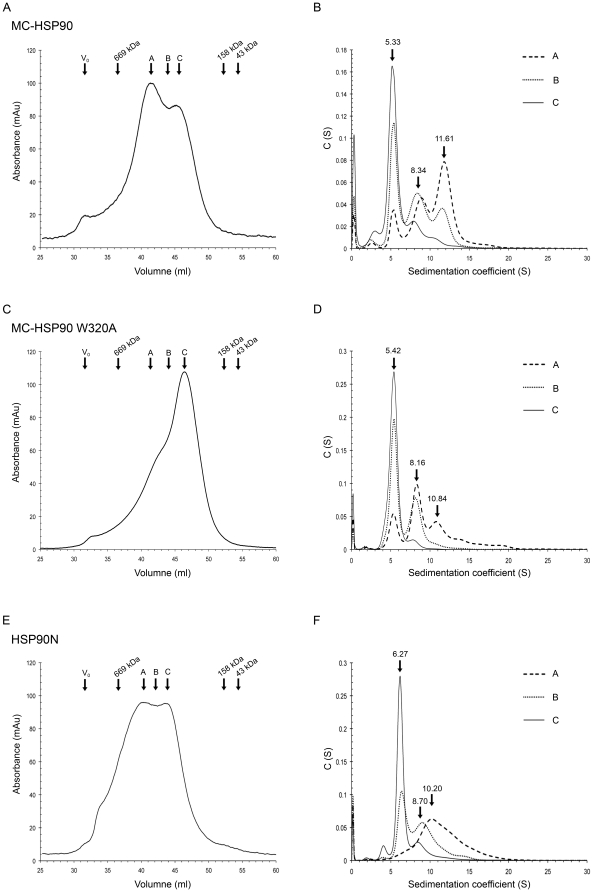
Oligomer states of MC-HSP90 and HSP90N. (A) (C) (E) The elution profiles of MC-HSP90, the mutant W320A, and HSP90N were recorded using size exclusion chromatography column S300. The void volume (V_0_) and retention volume of protein molecular weight standards and the positions of peaks (peak A and peak B) and overlap regions (region C) of proteins are shown above the curves. (B) (D) (F) The sedimentation coefficient distribution profiles of the protein samples from peak A, peak C, and the overlap region (B) from S300. The predicted sedimentation coefficients by SEDFIT for dimer, tetramer, and hexamer of MC-HSP90, W320A and HSP90N are indicated.

In order to understand the oligomeric states of MC-HSP90 in solution, size-exclusion chromatography and sedimentation velocity (SV) experiments were carried out to determine their molecular size. The elution profile of size-exclusion chromatography for MC-HSP90 shows two major peaks between the protein marker thyroglobulin (669 kDa) and aldolase (158 kDa). It suggests that MC-HSP90 contains at least two oligomeric states (Figure 6A). No corresponding peak for the monomer was observed, indicating that the monomeric species was not present or not detectable under our experimental conditions. Based on the elution profile, the two peaks were calculated to have molecular masses of about 252 and 408 kDa corresponding to approximately five and eight subunits, respectively. However, MC-HSP90 is not a spherically shaped globular protein and it is difficult to predict the molecular mass of its oligomeric states by size exclusion chromatography, especially when it contains a flexible C-terminal tail. To obtain further information of the oligomer state, the peak fractions and those in the overlap region eluted from the size exclusion chromatography were analyzed separately by analytical ultracentrifugation. Surprisingly, the continuous *c(s)* distribution suggests that MC-HSP90 exists as three species in solution with sedimentation coefficients of 5.3S, 8.3S and 11.6S (Figure 6B), with a frictional ratio of 1.3. The calculated masses of 94.4 kDa, 189.6 kDa and 299.1 kDa, corresponding to dimer, tetramer and hexamer, correlate very well with the actual molecular weights.

**Figure 7:**
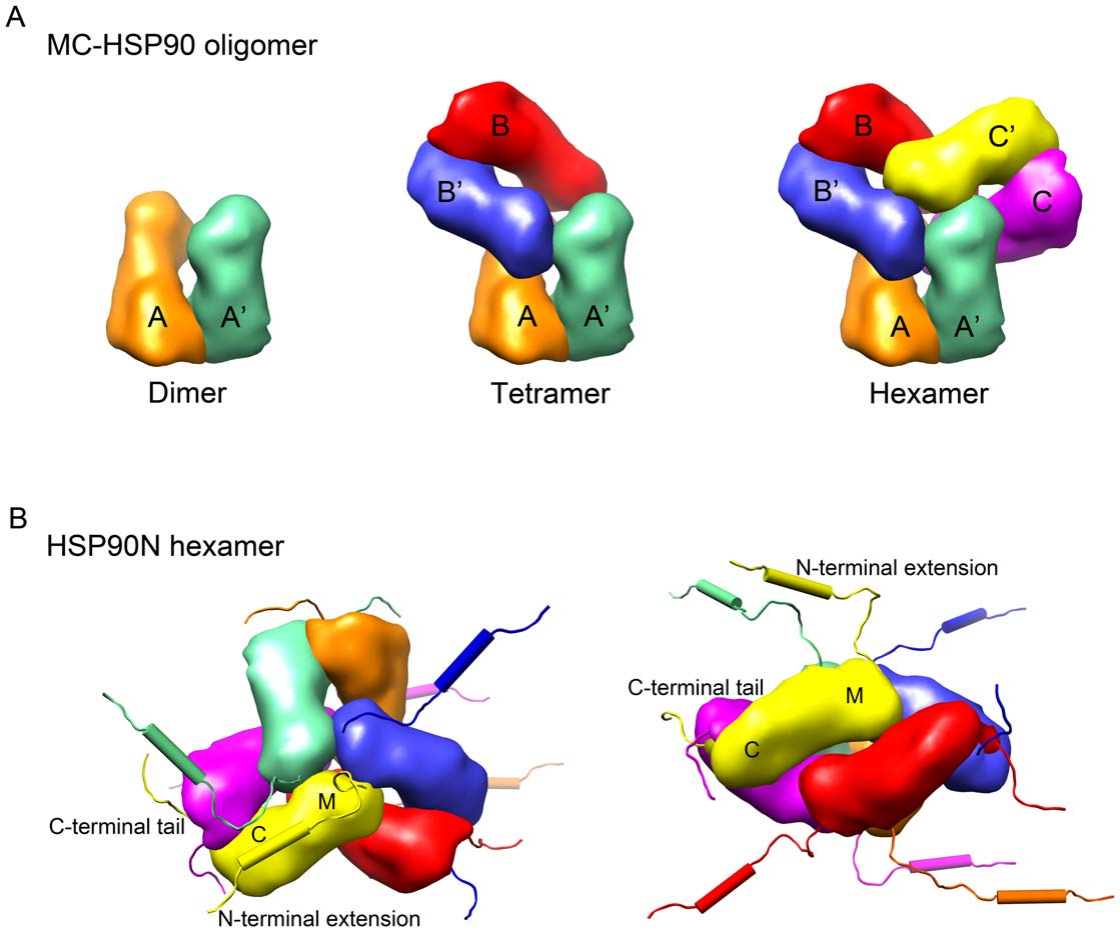
MC-HSP90 assembly and models of HSP90N hexamer. (A) Assembly of MC-HSP90 hexameric structure. The process begins with a monomer (orange) subunit associating with another monomer (green) to form a stable dimer as the building block. It continues with further association of dimers via the N-terminal interface of M domain to form a possible dimer of dimers (or tetramer) and a stable hexamer. The occurrence of tetramer might represent an intermediate. In the models of MC-HSP90 dimer, tetramer and hexamer shown here, the building blocks are paired and indicated by the labels AA′, BB′ and CC′. (B) Top view and side view of HSP90N hexamer with 12 flexible polypeptide segments hanging around the core structure. The protomers of hexameric HSP90N are represented by different colors, green, orange, blue, yellow, red, and megenta. The N-terminal hydrophilic extension and C-terminal tail of the yellow protomer are labeled.

Based on the observed 3D structure of MC-HSP90 determined in the *P*2_1_ crystal, the sedimentation coefficient values of MC-HSP90 at dimer and each oligomer states were calculated by using the computer program HYDROPRO [22], and compared with the experimental values from the sedimentation velocity experiments. As shown in Figure 7A, the atomic models of monomer, dimer, tetramer and hexamer yielded sedimentation coefficients of 3.7S, 5.7S, 8.8S and 11.9S, respectively. These HYDROPRO predicted S values for MC-HSP90 dimer, tetramer and hexamer models were similar to the experimental results (Table 2). The agreement between the calculated and experimental values confirms the existance of MC-HSP90 in these oligomeric states. Furthermore, during the gel-filtration separation and sedimentation velocity experiments, the MC-HSP90 oligomers did not undergo rapid interconversion between one another. Otherwise the redistribution of protein in different states would result in similar pattern of the sedimentation velocity profiles, for some equilibrium state. It indicates that the association and dissociation between each state is irreversible or the equilibrium rate is very slow due to large energy barriers.

**Table 2:** Sedimentation coefficient results and molecule weights.

	MC-HSP90			MC-HSP90 W320A		HSP90N	
	*S* a	*S*	MW^b^	*S*	MW^b^	*S*	MW^b^
Dimer	5.71	5.33	94.4	5.42	92.6	6.27	118.8
Tetramer	8.92	8.34	189.6	8.16	185.0	8.70	205.0
Hexamer	11.87	11.61	299.1	10.84	278.6	10.20	280.4

a The S values predicted by atomic coordinates model of MC-HSP90 using the software HYDROPRO.

b The calculated molecular weights by sedimentation velocity experiments.

As shown above, the residue W320 may play an important role in the hexamer assembly. Here it was mutated into an alanine and the resulting mutant protein W320A of MC-HSP90 was subjected to similar analyses as was the wild-type protein for its molecular size distribution. The size-exclusion chromatography profile showed a large shift of the oligomeric states toward the dimer (Figure 6C). The subsequent SV analyses showed sedimentation coefficients of 5.4S, 8.2S and 10.8S for three major oligomer states, with corresponding molecular mass of 92.6 kDa, 185.0 kDa and 278.6 kDa for dimer, tetramer and hexamer, respectively (Figure 6D and Table 2).

### Oligomer States of HSP90N

The HSP90 isoform HSP90N lacks the N-terminal ATPase domain, which is replaced by a 30-residue segment, but the other related domains are the same as HSP90α. HSP90N has the same N-terminal hydrophilic region (residues 31–100), the middle client protein binding domain, and the C-terminal dimerization domain as does HSP90α. Thus the hexameric structure of MC-HSP90 should somewhat resemble the quaternary structure of HSP90N. To test this hypothesis, size exclusion chromatography and sedimentation velocity experiments were also conducted on HSP90N. The size-exclusion chromatography elution profile showed two broad peaks with molecular weight of about 309 and 473 kDa (Figure 6E). When the elution profile was compared with that of MC-HSP90, it shows that the apparent molecular sizes in both peaks are larger than those of the corresponding oligomers of MC-HSP90. Protein solutions collected from the two peaks and the overlap region were further analyzed for their sedimentation coefficients. The sedimentation velocity profiles of the low molecule weight peak and the overlap region show two states with sedimentation coefficients of 6.3S and 8.7S have calculated masses of about 118.8 kDa and 205.0 kDa (Figure 6F), and have a frictional ratio of 1.3, which correspond to the dimer and tetramer of HSP90N. However, the sample from the high molecule weight peak has a broad distribution at SV analysis with a major S value of 10.2S and has a frictional ratio of 1.30. The S value is lower than the MC-HSP90 hexamer and corresponds to a calculated mass of 280.4 kDa (Table 2). If the frictional ratio of 1.55 was used to analyze the S value of three oligomer states, it yielded 6.32S, 8.43S and 10.43S for dimer, tetramer and hexamer with molecular mass of 153.0, 267.4 and 371.7 kDa, respectively. We surmise that the N-terminal 100 amino acid unstructural region can endow HSP90N with a loose shape in the hexameric state due to the flexible nature of the extra N-terminal segment, and cause the density to decrease upon the formation of HSP90N hexamer. The sedimentation velocity experiments also showed that HSP90N exists in similar dimer and tetramer states as does MC-HSP90.

### The 3D models of hexameric HSP90N

The N-terminal extension region of HSP90N comprising of residues 1–100 contains a hydrophobic helix, which was predicted as a transmembrane helix by three web version prediction programs. (See Materials and Methods for details.) The results are highly consistent, though different prediction methods yielded slight variations in the start and end points for the transmembrane segment. According to the prediction results of the HMMTOP program, the segment contains a transmembrane helix of 19 amino acids at residues 12–30 (Figure 4A). Based on the above conclusions derived from the sedimentation velocity experiments and the predictions of transmenbrane helix, a model was constructed for the quaternary structure of HSP90N. In the model (Figure 7B), six protomers of HSP90N with N-terminal extension and C-terminal tail are assembled into a hexameric structure, which has an architecture of triangular bipyramid geometry as was observed for the MC-HSP90 hexamer. The core structure is built by middle and C-terminal domain comprise residues 294 to 695 from the six protomers, The N-terminal extensions with the transmembrane helix are extended from the N-terminus of M domain around the cental 3-fold axis, three segments facing up and the other three facing down. The C-terminal tails extend from the dimerization domain toward the rim of the hexamer.

## Discussion

It is well established that HSP90 C-terminal domain is required for its dimerization [21], and the dimer is the building block of its oligomeric structures [18], [23]. On the structural level, the HSP90 oligomerization process was not clearly understood. In this study, we describe the hexameric structures of N-terminal truncated human HSP90, MC-HSP90 and MC-HSP90-Ct crystallized in four crystal forms, *P*2_1_, *C*222_1_, *R*32 and *P*6_3_22, which have six, three or one molecules in their asymmetric unit. The protein molecules were assembled into a virtually identical hexameric structure with triangular bipyramid geometry in all crystal forms. Other similar hexameric architecture had been observed in the crystal structures of N-terminal truncated yeast HSP82 (PDB: 2CGE) and *Leishmania major* HSP83-1 (PDB: 3HJC), which have rmsd of 1.167 Å for 262 atoms (Figure 3D and Video S1) and 1.066 Å for 319 atoms when compared with the MC-HSP90 hexamer (*P*2_1_ crystal form), respectively. However, this kind of hexamer structure was not present in the crystal structure of N-terminal truncated GRP94 (PDB: 2O1T). The triangular-bipyramid hexamer is assembled by using six MC-HSP90 molecules. The C-terminal dimerization and the interactions from the M domain contact make the formation of MC-HSP90 hexamer; each protomer provides the interface of about 1267 Å^2^ and 531 Å^2^ for the dimerization of MC-HSP90 monomer and trimerization of MC-HSP90 dimer. Presumably dimerization occurs prior to trimerization for hexamerization. The residues W320 and R346 play the key roles for associating MC-HSP90 dimers into the hexamer. These two residues are conserved in other 90-kDa heat shock proteins, including yeast HSP82 and *Leishmania major* HSP83-1, but not found in canine GRP94, these two redidues are Ser in GRP94 (Figure 4B and 4C).

In fact, W320 of human HSP90, equvalent to W300 of yeast HSP82, was found to be required for interaction with the client protein PKA/AKt [24]. W320 packs against the W19 and Y23 of Aha1 in the M-HSP90-N-Aha1 complex structure [25], and the binding site was involved in N and M domain conmunication in yeast HSP82 with similar interactions (Figure 5). The aromatic side chain of F200 in the N-terminal domain has hydrophobic interactions with the pocket formed by T273, P275, W277, F292 and Y344 in the middle domain [3]. These residues in the 90-kDa heat shock chaperones are hightly conserved in many species (Figure 4C). In human HSP90, the hydrophobic pocket formed by P295, W297 and F312 may allow binding to the residue F213 (conserved as F200 in yeast HSP82) for the N and M domain communication. In addition, the pocket provides a specific concave site for MC-HSP90 oligomerization. That the mutant W320A showed a shift toward dimer formation but the tetramer and hexamer did not disappear suggests the conservation of an intact hydrophobic pocket, which can still interact with the alanine side chain, although in a significantly weaker manner.

Hydrodynamic analysis indicated that MC-HSP90 has three oligomer states in solution. The crystal structures and the program HYDROPRO predictions indicate that the three states are dimer, tetramer and hexamer. These even-numbered oligomers are the main species observed. The architecture for each oligomer states of MC-HSP90 are shown in Figure 6A. In the oligomerization process, two MC-HSP90 molecules associated into a stable dimer by their C-terminal domain. The dimer structure is the building block of MC-HSP90 oligomers. Two MC-HSP90 dimers can associate into a tetramer by their N-terminal contacts of M domain. After the tetramer intermediate state is formed, the third dimer can insert into the tetramer to form the complete hexamer with triangular bipyramid geometry. HSP90 oligomerizes into tetramers and hexamers at high temperature, and the even-numbered oligomers seem to be active in binding and chaperoning unfolded proteins [15]. HSP90 existing as oligomers in the cytosol had been demonstrated, and that MC-HSP90 dimer oligomerizes into tetramer and hexamer is in agreement with previous reports. The truncated form carrying residues 290–732 of HSP90α tended to form oligomers even without heat treatment, and the C-terminal 200 amino acids were capable of forming oligomers [14]. The heat-induced oligomerization occurs upon the heat treatment at 50°C, and ATP and ADP inhibit the oligomerization by stabilizing a dimeric structure of HSP90 that is less sensitive to heat [23]. High temperatures usually increase the entropy and promote the hydrophobic interactions between proteins. It has been pointed out that HSP90 oligomerization at high temperature may be driven by hydrophobic interactions [23]. The hydrophobic contacts as shown above between the side-chains of non-polar residues in the M domain may represent one of the specific temperature-sensitive interactions to modulate the oligomerization of HSP90.

Lacking the ATPase domain, two MC-HSP90 molecules are associated into an “open” form dimer by its C-terminal helices (residues 640–695), and the C-terminal tail truncated MC-HSP90-Ct in the hexagonal crystal also has the open-form dimer (data not shown). The open-form dimer is required for hexamer packing and is responsible for the ATPase-coupled molecular clamp mechanism of HSP90 chaperone [3], whose opening and closing by transient N-terminal dimerization are directly coupled to the ATPase cycle [2], and also involves the co-chaperone p23/Sba1 recruitment [3]. The N domain and M domain of HSP90 are separated by a flexible link of about 60 residues, thus the interactions between these two domains are essential for the formation of close-form HSP90. The linker allows N-domain movements for different intersubunit interactions.

HSP90N also shows three oligomer states in solution. It lacks the N-terminal ATPase domain and contains the amino acids 224–732 of HSP90α. But it retains the linker and has an additional hydrophobic segment at its N-terminus. Based on the crystal structure and hydrodynanic properties of MC-HSP90, the three oligomer forms should be the dimer, tetramer and hexamer and have similar quaternary structures to MC-HSP90. The hexameric structure of HSP90N reconstructed by modeling is shown in Figure 7. HSP90N hexamer has a similar core structure as MC-HSP90, but the 12 flexible segments of additional N-terminal (100 residues) and C-terminal tails (35 residues) make it a disheveled sphere, resulting in the decrease of protein density and sedimentation coefficients in the hydrodynamic analysis. In the present study of MC-HSP90 and HSP90N, it is shown that in the lack of ATPase domain HSP90 self-oligomerizes into a hexameric structure, and the presence of N-terminal transmembrane segment does not affect the oligomerization of MC domains at room temperature. Heat-induced oligomerization is affected by the modulators ATP and ADP that are known to bind to the ATPase domain and affect HSP90 function [23]. Thus, the ATPase domain may play a central role in the heat-induced oligomer dissociation for its chaperone function.

In summary, we have determined the crystal structures of middle and C-terminal domain (MC-HSP90) of human HSP90α. The structure reveals a hexameric assembly with triangular bipyramid geometry, in which the building block is a dimer. In addition to the four-helix bundle formed by the C-terminal dimerization domain, the residue W320 and the hydrophobic pocket in the middle domain provide the major interactions for the oligomerization. Size exclusion chromatography and analytical ultracentrifugation experiments indicated that MC-HSP90 not only forms the functional dimer, but also exists in tetrameric and hexameric states in solution. HSP90N in the dimer, tetramer and hexamer states were also elucidated here. The hexamer structure of MC-HSP90 presented here illustrates the possible oligomer structures of HSP90N. For cancer therapies, inhibitor/drug development has been focused on inhibition of the N-terminal ATPase domain of HSP90, such as the purine mimetic PU24FC1, geldanamycin (GA) and 17-allylamino-17demethoxygeldanamycin (17-AAG) [26]. Here, the hexameric structure of MC-HSP90 reveals the possible oligomeric states of HSP90 for its chaperone activity. The interaction sites at oligomerization and domain communications could be the new targets for structure based design of anti-cancer drugs to inhibit the HSP90 and HSP90N activity.

## Materials and Methods

### Preparation of MC-HSP90, MC-HSP90-Ct, and HSP90N

For HSP90 production, a pCMV6-XL5 plasmid (OriGene Technologies, Inc) containing a full length cDNA of the human heat shock protein 90α gene (NM_005348.2) was used as the initial DNA template. The DNA fragments of HSP90 with an N-terminal Factor Xa cleavage site and a C-terminal His-tag were generated using PCR amplification with primers 5′-CGCGGATCCATTGAGGGTCGCGCTGCTATGCCTGAGGAAACCC-3′ (forward, encoding the Factor Xa cleavage site and two extra Ala residues (underlined)) and 5′-CCGCTCGAGT TAGTGATGGTGATGGTGATGGTCTACTTCTTCCATG-3′ (reverse, encoding the C-terminal His-tag (underlined)), and then cloned into a pGEX-6p-1 vector (GE Healthcare) at the *BamH* I and *Xho* I sites. This full length HSP90 construction (residues 1–732), termed pGEX-6p-1-HSP90, was used as the DNA template for making the plasmids of MC-HSP90 (pGEX-6p-1-MC-HSP90), MC-HSP90-Ct (pGEX-6p-1-MC-HSP90-Ct) and HSP90N (pGEX-6p-1-HSP90N). The gene encoding MC-HSP90 (residues 293–732) and MC-HSP90-Ct (residues 293–697) were cloned using the same forward primer (5′-CGCGGATCCATTGAGGGTCGCGCTGCTACAAAGCCCATCTGG-3′) which also encodes a Factor Xa cleavage site and two extra Ala residues. The HSP90N plasmid (pGEX-6p-1-HSP90N) was constructed by overlap PCR method, using six forward primers (5′-TTATTTTCTTTATTGGAGTTATTTTAAGGAACGTGATAAAGAAGTAAGCGATGATGAGGC-3′, 5′-GTAAAGTTGAATGTAGTTTTCTTTATTTTCTTTATTGGAGTTATTTTAAGGAACG-3′, 5′-GTAATTGTAATTTTTGTGTTAGTAAAGTTGAATGTAGTTTTCTTTATTTTCTTTATTGG-3′, 5′-GGGTGAAAAAGCAAGTTATTGTAATTGTAATTTTTGTGTTAG-3′, 5′-CGCGCTGCTATGGGTGAAAAAGCAAGTTATTGTAATTG-3′ 5′-CGCGGATCCATTGAGGGTCGCGCTGCTATGGGTGAA-3′) to synthesize the DNA encoding the Factor Xa cleavage site, the extra Ala residues, and the 30 N-terminal amino residues of HSP90N. In both constructs of pGEX-6p-1-MC-HSP90 and pGEX-6p-1-HSP90N, the pGEX-6p-1-HSP90 plasmid was used as the initial template, and a reverse primer, 5′-CCGCTCGAGT TAGTGATGGTGATGGTGATGGTCTACTTCTTCCATG-3′ was used. For pGEX-6p-1-MC-HSP90-Ct a different reverse primer of 5′-CCGCTCGAGTTAATGGTGATGATGGTGATGGTGATGACCCAGACCAAG -3′ was used. Mutagenesis of MC-HSP90 W320A was made using the Quick-change site directed mutagenesis kit (Stratagene) on the recombinant plasmid pGEX-6p-1-MC-HSP90 with the following pair of oligonucleotides: forward, 5′-GAG CTTGACCAATGACGCTGAAGATCACTTGGCAG-3′; reverse, 5′-CTGCCAAGTGATCTTCAGCGTCATTGGTCAAGCTC-3′.

Each of the HSP90 plasmids (pGEX-6p-1-MC-HSP90, pGEX-6p-1-MC-HSP90-Ct, and pGEX-6p-1-HSP90N) was transformed separately into *Escherichia coli* BL21 (DE3) cell (Novagen) for protein overexpression. MC-HSP90 protein was pre-purified by chromatography using a nickel-nitrilotriacetic acid (Ni-NTA) column (Qiagen) and a glutathione column (GSTrap FF, GE Healthcare). The glutathione S-transferase (GST) -tagged protein was digested by Factor Xa protease at 4°C for 24 hours in 150 mM NaCl and 50 mM Tris, pH 8.0, and then the cleaved MC-HSP90 was separated by ion-exchange chromatography on a HiTrap™ 26/10 QFF column (GE Healthcare). The protein was eluted at 350–400 mM NaCl, 50 mM Tris-HCl, pH 8.0. Uncleaved MC-HSP90 and free GST were eliminated by flowing through a GSTrap FF column, and pure MC-HSP90 was collected in the flow-through fractions. The MC-HSP90-Ct and HSP90N proteins were purified by similar protocols, in which the first gluthathione column was omitted in both cases and the QFF column was replaced by a second Ni-NTA column for MC-HSP90-Ct. The mutant protein W320A was expressed and pufiried as was the wild-type MC-HSP90. The purified proteins were then dialyzed against a solution of 50 mM Tris-HCl, pH 8.0, 1 mM EDTA and 1 mM dithiothreiol (DTT), and then concentrated using a 30 kDa filter (Amicon Ultra filter, Millipore) to about 12 mg/ml for stock at −80°C.

### Crystallization and Data Collection

The crystals of MC-HSP90 were grown by the sitting-drop vapor diffusion method. A solution of 2.5 M ammonium sulfate, and 0.1 M Tri-sodium citrate at pH 5.4–5.8 was used as the reservoir. Three crystal forms *C*222_1_, *P*2_1_ and *R*32 were obtained using different crystallization droplet preparations. For the *C*222_1_ and *P*2_1_ crystals, the protein solutions were pre-incubated with solutions containing the platinum additive *cis*-dichloro(ethylenediamine)platinum (II) and cisplatin, respectively, at a molar ratio of 1∶2 for one hour at 20°C. The mixed protein-Pt solutions (1.5 µl) were then combined with the reservoir (1.0 µl) for crystallization droplet set-up. The crystallization droplet of *R*32 crystal were prepared by mixing 1.5 µl of the protein solution with 1 µl reservoir solution and 0.5 µl additive solution, Silver Bullets No. 18 which contains 0.33% w/v 2,6-Naphthalenedisulfonic acid disodium salt, 0.33% w/v 2-Aminobenzenesulfonic acid, 0.33% w/v m-benzenedisulfonic acid disodium salt, and 0.02 M HEPES sodium pH 6.8 (Hampton Research). MC-HSP90-Ct crystals were crystallized in the *P*6_3_22 space group, with a reservoir solution of 2.6 M ammonium sulfate, 0.1 M Tri-sodium citrate at pH 5.0 and the additive solution of Silver Bullets No. 70 (0.2% w/v anthrone, 0.2% w/v benzidine, 0.2% w/v N-(2-acetamido)-2-aminoethanesulfonic acid, 0.2% w/v phenylurea, 0.2% w/v β-Alanine, 0.02 M HEPES sodium pH 6.8) (Hampton Research). All crystals were flash-frozen with 13–15% glycerol (vol/vol) as a cryo-protectant and the diffraction patterns were recorded at cryogenic temperatures. The diffraction data of *C*222_1_, *P*2_1_ and *R*32 crystals of MC-HSP90 were collected at the wavelength of 1.000 Å from the synchrotron beamline NW12 of Photon Factory (PF) in Japan, using an ADSC Quantum-210 charge-coupled diode (CCD) detector. Data of the *P*6_3_22 crystal of MC-HSP90-Ct were collected at the wavelength of 0.900 Å from the synchrotron beamline BL44XU of SPring-8 in Japan, using an MX-225 CCD detector. All data were processed and scaled using the program HKL2000 [27].

### Structure Determination and Refinement

The *C*222_1_ crystal structure was determined by molecular replacement using the program Molrep of the CCP4 program suite [28] and the structure of yeast HSP82 MC domains (2CGE) was used as a search model. The orthorhombic crystal form has three molecules in an asymmetric unit.

The *P*2_1_ and *R*32 crystal structures of MC-HSP90 and the *P*6_3_22 crystal structure of MC-HSP90-Ct were solved using Molrep, with the partially refined *C*222_1_ crystal structure as the search model. The monoclinic crystal form has six monomers in an asymmetric unit, and both rhombohedral and hexagonal crystal forms have one molecule in an asymmetric unit.

Throughout the refinement, 5% of randomly selected data were set aside for cross validation with *R*free values. Manual modifications of the models were performed using the program XtalView [29] and Coot [30]. Difference Fourier (Fo-Fc) maps were calculated to locate the solvent molecules.

The *C*222_1_ crystal structure was refined for individual atomic positions and temperature factors, and the model yielded *R*_work_/*R*_free_ values of about 22/27 using the program Crystallography and NMR System (CNS) version 1.2 [31]. The models derived from the CNS refinement were further refined using Refmac5 [32], including individual isotropic B-factor refinement and translation-libration-screw (TLS) refinement [33] and yielding *R*_work_/*R*_free_ values of 20.9/25.2.

The monoclinic crystal structure was refined with strong NCS restraints and refined to *R*_work_/*R*_free_ values of 24/30 for all data at 3.05 resolution. After addition of solvent molecules, NCS restraints were removed and the model was refined for individual atomic positions and temperature factors, yielding *R*_work_/*R*_free_ values of about 23/28 using the program CNS. The model was further refinemet using Refmac5, including individual isotropic B-factor refinement and TLS refinement, from which the *R*_work_/*R*_free_ values of 20.4/25.9 were obtained.

Data collection and final model statistics are shown in Table 1. The molecular figures were produced by using UCSF Chimera [34]. The atomic coordinates and structure factors of the *C*222_1_ and *P*2_1_ crystal structures have been deposited in the Protein Data Bank with accession codes 3Q6M and 3Q6N, respectively.

### Size Exclusion Chromatography

The protein solutions (100 µl) of MC-HSP90 and HSP90N at final concentrations of 7–10 mg/ml were loaded separately onto a Sephacryl S-300 HR (GE Healthcare) size exclusion chromatography column of 1 cm×120 cm (Econo-column, Bio-Rad). The column was equilibrated with a buffer solution of 50 mM Tris-HCl, pH 8.0, and eluted at a flow rate of 0.5 ml/min at room temperatures. OD_280_ was monitored for the eluted solution. The elution volume corresponding to each oligomer types were then selected for analytical ultracentrifugation analysis. Thyroglobulin (669 kDa), aldolase (158 kDa) and ovalbumin (43 kDa) included in the Gel Filtration Calibration Kits (GE Healthcare) were used as the markers to estimate the molecular weight.

### Analytical Ultracentrifugation

Sedimentation velocity (SV) experiments were performed at 45,000 rpm using a 4-hole AnTi60 rotor at 20°C in a Beckman Optima XL-I AUC equipped with absorbance optics. The MC-HSP90 and HSP90N samples collected from the gel-filtration column were diluted to a final concentration of 0.3 mg/ml using 100 mM Tris-HCl buffer. Standard 12-mm aluminum double-sector centerpieces were filled with protein solution, and the reference cell contained the blank buffer. Quartz windows were used with absorbance optics (OD_280_) in a continuous mode without averaging. No time interval was set between scans. Data were analyzed with a *c(s)* distribution of the Lamm equation solutions calculated by the program SEDFIT Version 12.1b (http://analyticalultracentrifugation.com). The software Sednterp (http://www.jphilo.mailway.com) was used to estimate protein partial specific volume (*Vbar*), buffer density (0.99966 g/ml) and buffer viscosity (0.010167 poise) at 20°C. The *Vbar* value of MC-HSP90 is 0.7375 ml/g, and that for HSP90N is 0.7342 ml/g. For the sedimentation coefficient (S) values predictions of MC-HSP90 oligomer states, the atomic coordinate models of MC-HSP90 dimer, tetramer and hexamer were extracted from the *P*2_1_ crystal structure and were used to calculate the S values by the software HYDROPRO, in which all user adjustable parameters were left at the default values [22].

### In-gel Protein Digestion and Mass Spectrometry

The protein bands on a 1D SDS-PAGE gel were manually excised to dehydrate with acetonitrile for 10 mins, vacuum dried, rehydrated with 55 mM DTE in 25 mM ammonium bicarbonate, pH 8.5, at 37°C for 1 h, and subsequently alkylated with 100 mM iodoacetamide in 25 mM ammonium bicarbonate, pH 8.5, at room temperature for 1 h. The pieces were then washed twice with 50% acetonitrile in 25 mM ammonium bicarbonate, pH 8.5 for 15 min each time, dehydrated with acetonitrile for 10 min, dried, and rehydrated with a total of 25 ng of sequencing grade, modified trypsin (Promega, Madison, WI) in 25 mM ammonium bicarbonate, pH 8.5, at 37°C for 16 hr. Following digestion, tryptic peptides were extracted twice with 50% acetonitrile containing 5% formic acid for 15 mins each time with moderate sonication. The extracted solutions were pooled and evaporated to dryness under vacuum. For MALDI-MS, the samples were pre-mixed 1 ∶ 1 with a matrix solution (5 mg/ml cyano-4-hydroxycinnamic acid in 50% acetonitrile, 0.1% v/v TFA, and 2% w/v ammonium citrate) and spotted onto a 96-well format MALDI sample plate. The data-directed obtain on the quadrupole time-of-flight (Q-TOF) Ultimat MALDI instrument.

### Membrane Topology Prediction and 3D Modeling

The amino acid sequence for HSP90N (ACCESSION: AAC25497) was downloaded from GenBank (http://ncbi.nlm.nih.gov). The web-versions of three different topology prediction methods were used to predict the topology of N-terminal residues 1–100 of the HSP90N protein: HMMTOP (http://www.enzim.hu/hmmtop/), TMHMM (http://www.cbs.dtu.dk/services/TMHMM-2.0/), and TMpred (http://bioweb.pasteur.fr/seqanal/interfaces/toppred.html). All these methods were used in the single sequence mode and all user adjustable parameters were left at their default values. Graphics of the 3D models of the hexameric HSP90N was produced with UCSF Chimera [34] using the solved MC-HSP90 hexamer structure of the *P*2_1_ crystal as a template.

## Supporting Information

Video S1 **Hexamer structural comparison.** The video shows the superposition of human MC-HSP90 hexamer and the N-terminal truncated yeast HSP82 (PDB: 2CGE) hexamer. The protomers of human MC-HSP90 in the same dimer are colored in blue and orange, and all protomers of yeast HSP82 are colored in green.
(MOV)

## References

[1] Trepel J, Mollapour M, Giaccone G, Neckers L (2010). Targeting the dynamic HSP90 complex in cancer.. Nat Rev Cancer.

[2] Prodromou C, Panaretou B, Chohan S, Siligardi G, O'Brien R (2000). The ATPase cycle of Hsp90 drives a molecular ‘clamp’ via transient dimerization of the N-terminal domains.. EMBO J.

[3] Ali MM, Roe SM, Vaughan CK, Meyer P, Panaretou B (2006). Crystal structure of an Hsp90-nucleotide-p23/Sba1 closed chaperone complex.. Nature.

[4] Mahalingam D, Swords R, Carew JS, Nawrocki ST, Bhalla K (2009). Targeting HSP90 for cancer therapy.. Br J Cancer.

[5] Powers MV, Clarke PA, Workman P (2009). Death by chaperone: HSP90, HSP70 or both?. Cell Cycle.

[6] Schweinfest CW, Graber MW, Henderson KW, Papas TS, Baron PL (1998). Cloning and sequence analysis of Hsp89alpha DeltaN, a new member of theHsp90 gene family.. Biochim Biophys Acta.

[7] Powers MV, Workman P (2007). Inhibitors of the heat shock response: biology and pharmacology.. FEBS Lett.

[8] Suzuki S, Kulkarni AB (2010). Extracellular heat shock protein HSP90beta secreted by MG63 osteosarcoma cells inhibits activation of latent TGF-beta1.. Biochem Biophys Res Commun.

[9] Scheufler C, Brinker A, Bourenkov G, Pegoraro S, Moroder L (2000). Structure of TPR domain-peptide complexes: critical elements in the assembly of the Hsp70–Hsp90 multichaperone machine.. Cell.

[10] Donnelly A, Blagg BS (2008). Novobiocin and additional inhibitors of the Hsp90 C-terminal nucleotide-binding pocket.. Curr Med Chem.

[11] Milicevic Z, Bogojevic D, Mihailovic M, Petrovic M, Krivokapic Z (2008). Molecular characterization of hsp90 isoforms in colorectal cancer cells and its association with tumour progression.. Int J Oncol.

[12] Grammatikakis N, Vultur A, Ramana CV, Siganou A, Schweinfest CW (2002). The role of Hsp90N, a new member of the Hsp90 family, in signal transduction and neoplastic transformation.. J Biol Chem.

[13] Zurawska A, Urbanski J, Bieganowski P (2008). Hsp90n - An accidental product of a fortuitous chromosomal translocation rather than a regular Hsp90 family member of human proteome.. Biochim Biophys Acta.

[14] Nemoto T, Sato N (1998). Oligomeric forms of the 90-kDa heat shock protein.. Biochem J.

[15] Yonehara M, Minami Y, Kawata Y, Nagai J, Yahara I (1996). Heat-induced chaperone activity of HSP90.. J Biol Chem.

[16] Nemoto TK, Ono T, Tanaka K (2001). Substrate-binding characteristics of proteins in the 90 kDa heat shock protein family.. Biochem J.

[17] Csermely P, Schnaider T, Soti C, Prohászka Z, Nardai G (1998). The 90-kDa molecular chaperone family: structure, function, and clinical applications. A comprehensive review.. Pharmacol Ther.

[18] Moullintraffort L, Bruneaux M, Nazabal A, Allegro D, Giudice E (2010). Biochemical and biophysical characterization of the Mg^2+^-induced 90-kDa heat shock protein oligomers.. J Biol Chem.

[19] Jakob U, Meyer I, Bügl H, André S, Bardwell JC (1995). Structural organization of procaryotic and eucaryotic Hsp90. Influence of divalent cations on structure and function.. J Biol Chem.

[20] Dollins DE, Warren JJ, Immormino RM, Gewirth DT (2007). Structures of GRP94-nucleotide complexes reveal mechanistic differences between the hsp90 chaperones.. Mol Cell.

[21] Minami Y, Kimura Y, Kawasaki H, Suzuki K, Yahara I (1994). The carboxy-terminal region of mammalian HSP90 is required for its dimerization and function in vivo.. Mol Cell Biol.

[22] García De La Torre J, Huertas ML, Carrasco B (2000). Calculation of hydrodynamic properties of globular proteins from their atomic-level structure.. Biophys J.

[23] Chadli A, Ladjimi MM, Baulieu EE, Catelli MG (1999). Heat-induced oligomerization of the molecular chaperone Hsp90. Inhibition by ATP and geldanamycin and activation by transition metal oxyanions.. J Biol Chem.

[24] Sato S, Fujita N, Tsuruo T (2000). Modulation of Akt kinase activity by binding to Hsp90.. Proc Natl Acad Sci U S A.

[25] Meyer P, Prodromou C, Liao C, Hu B, Roe SM (2004). Structural basis for recruitment of the ATPase activator Aha1 to the Hsp90 chaperone machinery.. EMBO J.

[26] Koga F, Kihara K, Neckers L (2009). Inhibition of cancer invasion and metastasis by targeting the molecular chaperone heat-shock protein 90.. Anticancer Res.

[27] Otwinowski Z, Minor W (1997). Processing of x-ray diffraction data collected in oscillation mode.. Methods Enzymol.

[28] Bailey S (1994). The CCP4 suite: programs for protein crystallography. Acta Crystallogr.. D Biol Crystallogr.

[29] McRee DE (1999). XtalView/Xfit – a versatile program for manipulating atomic coordinates and electron density.. J Struct Biol.

[30] Emsley P, Cowtan K (2004). Coot: model-building tools for molecular graphics.. Acta Crystallogr D Biol Crystallogr.

[31] Brunger AT, Adams PD, Clore GM, DeLano WL, Gros P (1998). Crystallography and NMR system: a new software suite for macromolecular structure determination.. Acta Crystallogr D Biol Crystallogr.

[32] Murshudov GN, Vagin AA, Dodson EJ (1997). refinement of macromolecular structures by the maximunlikelihood method.. Acta Crystallogr D Biol Crystallogr.

[33] Winn MD, Isupov MN, Murshudov GN (2001). Use of TLS parameters to model anisotropic displacements in macromolecular refinement.. Acta Crystallogr D Biol Crystallogr.

[34] Pettersen EF, Goddard TD, Huang CC, Couch GS, Greenblatt DM (2004). UCSF Chimera–A visualization system for exploratory research and analysis.. J Comput Chem.

